# New Plastic Crack-Tip Opening Displacement Tool Based on Digital Image Correlation for Estimating the Fatigue-Crack-Growth Law on 316L Stainless Steel

**DOI:** 10.3390/ma16134589

**Published:** 2023-06-25

**Authors:** Muhammad Ajmal, Cristina Lopez-Crespo, Alejandro S. Cruces, Pablo Lopez-Crespo

**Affiliations:** 1 Department of Civil and Materials Engineering, University of Malaga, C/Doctor Ortiz Ramos, s/n, 29071 Malaga, Spainascruces@uma.es (A.S.C.); 2 Yanbu Industrial College, Yanbu 46452, Saudi Arabia; 3 Jesus Marin Polytechnic Institute, C/Politecnico, 1, 29007 Malaga, Spain

**Keywords:** crack-tip opening displacement, digital image correlation, fatigue crack propagation

## Abstract

This work presents a new approach for studying crack growth resulting from fatigue, which utilizes the plastic contribution of crack-tip opening displacement (CTOD_p_). CTOD_p_ is used to predict austenitic stainless-steel crack propagation. Unlike linear elastic fracture mechanics analysis, the method presented here is also helpful for tasks other than small-scale yielding. The approach was based on correlating full-field displacement information with post-processing digital images. This work describes a detailed post-processing protocol that can be used to calculate CTOD_p_. The results for steel compact-tension specimens were especially promising. Of note, there was a linear relationship between the propagation rate of fatigue cracks and the CTOD_p_ range.

## 1. Introduction

Engineers, designers, and researchers from all over the world face constant demand to reduce cost, weight, and emissions in engineering systems. When these systems are put into operation, most of their individual components experience cyclic stress from pressure changes, wind, waves, or vibrations. Consequently, the majority of system failures result from fatigue [1]. When designing elements, damage tolerance methods anticipate that there will be inherent flaws caused by manufacturing practices (e.g., faults in casting, machining, welding, and additive fabrication) [2] and unforeseen loading circumstances, thermal stresses, and fatigue [3]. During operation, these minor flaws can develop into larger cracks, primarily because of fatigue.

Fatigue is localized structural damage that worsens over time and causes liability issues, environmental contamination, and economic disruption [4]. Given that precise estimates of fatigue-crack-growth rates (FCGRs) are essential to defining the interval between maintenance inspections during crack growth, fatigue performance considerations are crucial in the design phase of new components and structures. In the literature, numerous models have been developed to estimate FCGRs based on different loads and material properties [5,6,7]. In this sense, Paris and Erdogan [8] proposed a model that employed a power law to relate the FCGR (da/dN) to the stress intensity factor (SIF) a as follows in Equation (1):

(1) $$\frac{da}{dN}=C{\left(\Delta K\right)}^{m}$$

*C* and *m* are constants that encompass material and environmental influences. Because there are analytical solutions [9,10,11] for a wide range of geometries, the aforementioned relationship has been exploited extensively. According to the premise that crack growth is controlled by the elastic field surrounding a crack [12], linear elastic fracture mechanics (LEFMs) remain advantageous for analyzing extensive cracks with small-scale yielding [8,13,14,15]. The SIF can also be useful for quantifying stress singularity according to the loading conditions and crack size. Nevertheless, the SIF has some limitations: (i) the process of obtaining da/dN–K relationships is purely empirical and was not developed based on the concepts of physics; and (ii) variable amplitude effects, the load ratio, and inconsistent behavior reported for short cracks cannot be explained [16].

In this sense, unlike crack-tip opening displacement (CTOD), the J-integral and the plastic zone size, which have physical significance and are easily understood, the K lacks of physical meaning because the K units were derived analytically based on the √r singularity. Thus, a number of alternatives have been proposed to avoid the limitations of the SIF, including the notion of crack closure, as suggested by Elber [17]. This proposal, which replaces ∆K with ∆K_eff_ (Equation (2)), slightly modified the Paris Law, which was founded on the assumption that the cycle phase representing the moment when the crack is fully closed should not be considered when estimating the growth rate of fatigue cracks.

(2) $$\frac{da}{dN}=C{\left(\Delta K_{eff}\right)}^{m}$$

Hence, $\Delta K_{eff}$ represents the SIF computed as the maximum load minus the opening load. Of note, the crack-closure levels also appear to be dependent on the measuring procedure. Even though closure was incorporated into the evaluation of short cracks [18], the stress state [19], specimen thickness [20,21], and load history [22], there is still some disagreement regarding the best method to determine the closure level. Moreover, some studies have suggested that the impact of crack closure resulting from plasticity is much lower when plane strain states dominate [23]. This strategy was expanded by incorporating the idea of partial crack closure [24,25], as well as other closure mechanisms, such as those produced by viscous fluids [26], phase changes [27], oxides [28], and roughness [29].

In contrast, other authors have suggested alternative methods that consider both the *K*_max_ and ∆*K* values as shields against crack growth [30,31]. Of note, the four-parameter Christopher–James–Patterson (CJP) and T-stress models used to understand the distribution of stresses in the vicinity of the crack tip [32,33] and the geometry effect [34,35,36,37], respectively, have also been used to investigate the FCGR. However, Ritchie and coworkers drew attention to the fact that the CJP model does not consider the connection between material properties and the FCGR [38].

While crack propagation appears to be an irreversible and non-linear process affecting the material surrounding the crack tip, the SIF range is an elastic and linear parameter. Thus, rather than the SIF itself, many researchers have focused on investigating fatigue crack growth based on the strain and stress fields [31], energy dissipated at the crack tip [39], cyclic plastic strain range [40,41], reversed plastic zone size [42,43], equivalent material concept [44,45], crack-opening displacement [46,47], strain range [48,49,50], and strain intensity factor [51]. In this context, the CTOD and the J-integral appear to be good candidates for including the plasticity effect in FCGR modelling [52]. 

Hence, Wells [53] initially proposed CTOD to explore the crack extension mechanism. This was further reinforced by the slip-based blunting mechanism, which indicated a clear connection between crack propagation and CTOD [46,54,55]. Subsequently, some authors postulated that the association between da/dN and CTOD is polynomial [56,57], while others suggested that the connection between CTOD and the rate of crack growth is linear [58]. Dong et al. [59] offered a model with a linear association between ∆CTOD and da/dN, while others [52,60] hypothesized a relationship between da/dN and the cyclic J-integral. Regardless, because CTOD is a local parameter, its experimental evaluation seems to be more convenient.

The determination of accumulated plastic strain [61], crack-tip strain fields [62], closure stress [63,64], plastic zone size [65], and effective values of ∆*K* [65,66,67] are just a few examples of how digital image correlation (DIC) has been successfully applied in the arena of fracture mechanics and fatigue in recent years. Part of the novelty of this present work was that CTOD was extracted from DIC-derived displacement fields from behind the crack tip. Antunes et al. first identified the utility of the range of the plastic CTOD (∆CTOD_p_) [68,69,70] given its strong influence on fatigue-crack-propagation processes. The aforementioned work was based on numerical data for different materials, but they more recently laid out a standard for differentiating between large- and small-scale yielding settings according to the contribution of the total elastic CTOD (CTOD_e_) [71]. Thus, the main purpose of this current research was the elucidation of a new crack-propagation law by experimentally determining ∆CTOD_p_ for 316L stainless steel.

## 2. Materials and Methods

**Figure 1:**
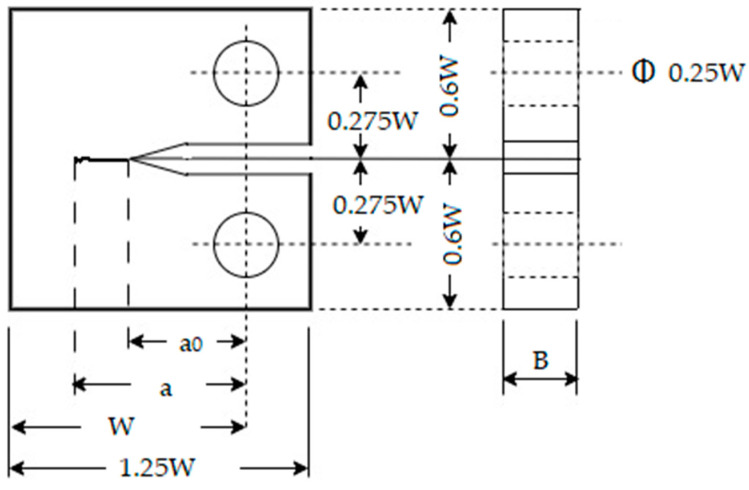
Schematic of the compact-tension (CT) geometry.

In this current work, 316L stainless steel was used. The Young’s modulus of E of this material is 195 GPa, with an approximate yield stress (σ_0_) of 304 MPa [64]. This alloy displays remarkable corrosion resistance and high strength and toughness even at subzero temperatures. As a result, it is widely employed in heavy gauge components for the shipbuilding, offshore, piping, transportation, aerospace, and nuclear industries [72]. An ASTM E64713 [73]-compliant compact-tension (CT) geometry-specimen configuration was used to fabricate a 50 mm wide fatigue-crack-growth specimen. All tests were conducted in one testing sample. The details of the geometry are given in Figure 1. Based on the relationship between the thickness and the specimen ligament, the specimen was deemed thick. A thickness of 12 mm, B, was used, with a 0.5 ratio between B and the length of the ligament. Electrical discharge machining (MV 1200-S M800 WIRE EDM, Mitsubishi Electric) was used to create an EDM notch in the CT specimen with a 90° opening angle and a notch-tip radius of about 0.25 mm.

### 2.1. Description of the Fatigue Tests

**Figure 2:**
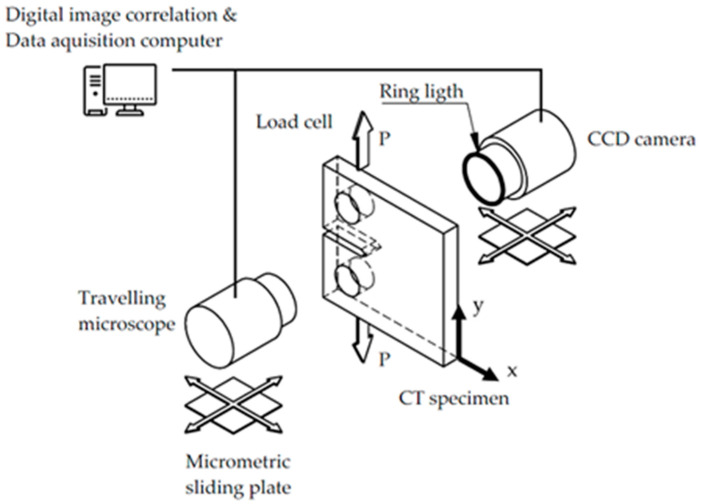
The experimental configuration employed in this work [64].

The critical stress intensity factor was K_c_ = 35 MPa$\sqrt{m}$ [64]. The fatigue-crack-growth experiments were conducted at 20 °C, employing a servo-hydraulic testing system from ESH Testing Ltd. (Brierley Hill, UK). With a loading range of ±10 kN. A total of 200,000 cycles were applied to pre-crack the specimen. Minimum and maximum loads of 0.15 and 2.95 kN, respectively, were used throughout the test. The DIC recording started at 230,792 cycles, with an initial crack length of 36.01 mm. The test finished at 331,273 cycles, with a final crack length of 37.35 mm. Four different fatigue stages were studied, as summarized in Table 1. Figure 2 shows a graphic representation of the experiment. The fatigue testing frequency applied to the specimen was 30 Hz, and the image acquisition required for DIC was carried out at several crack-growth stages. The frequency utilized for the load during image acquisition was substantially lower (1/100 Hz) than that applied for the rest of the experiment. 

### 2.2. Digital Image Correlation Technique

**Figure 3:**
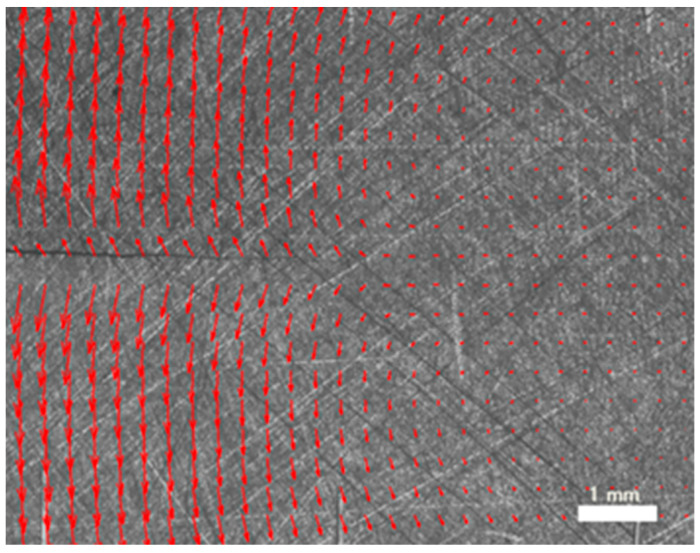
Photograph of the propagating crack on the compact-tension geometry specimen. The displacement field is shown as red directional arrows superimposed onto the image. The fatigue crack runs horizontally, growing from the left to right side.

Both image acquisition and image correlation processing were performed using LaVision (DAVIS StrainMaster) [74]. A macro lens with a teleconverter was fixed to a 12-bit 4-mega-pixel charge-coupled device (CCD) camera in the experimental optical setup (Figure 2). Even specimen surface lighting was achieved with lens-coupled ring illumination. To create a random texture that would provide enough contrast for the correlation method, the surface was sanded with medium-grit silicon carbide paper. Next, a 10 × 10 mm^2^ area was scanned with a 0.2 pixel per µm conversion factor. The displacement fields were generated using 32 × 32 pixel interrogation windows with a 75% overlap over the 200 frames captured during each loading cycle. The surface finish quality produced by the grit paper, as well as the distribution of the displacement vectors around the crack, are shown in Figure 3. Some additional details about the DIC technique used in this work can be found in previous publications [75,76].

## 3. Post-Processing Strategy

### 3.1. Identification of the Displacement of the Crack-Tip Opening 

**Figure 4:**
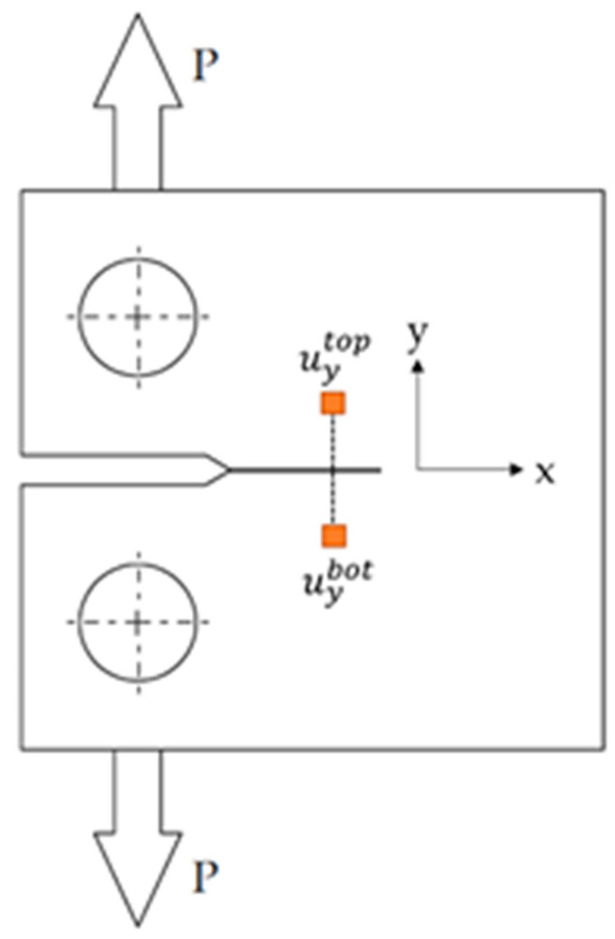
Description of data sets collected above the crack plane, $u_{y}^{top}$, and those collected below the crack plane, $u_{y}^{bot}$, used to compute displacement of the crack-tip opening.

The CTOD range (ΔCTOD) was determined at different positions from the tip by subtracting the vertical displacement data collected below the crack plane ($u_{y}^{bot}$, Figure 4) from the vertical displacement data collected above the crack plane ($u_{y}^{top}$, Figure 4), as also described in Equation (3) [64]:

(3) $$\Delta CTOD(x)=u_{y}^{top}-u_{y}^{bot}$$

where *x* is the direction of crack growth and *y* represents the direction of the crack widening, as shown in Figure 2 and Figure 4. The rigid body motion that may have occurred during loading was removed. Any experimental method that can provide full-field displacement information, including DIC [77], Moiré interferometry [78], and electronic speckle-pattern interferometry [66], can be employed to calculate the CTOD following the above description. Furthermore, Equation (3) can determine CTOD in both the scenario depicted in Figure 2 and in the case of a wedge-applied load. The crack-tip position was first identified from the horizontal and vertical displacement maps generated using DIC [79,80]. 

Of note, the distance behind the crack tip used to estimate CTOD also influenced the output values. In this work, CTOD was evaluated 104 µm (20 pixels) from the crack tip (see Figure 4) because, when detecting the crack tip as previously indicated, the pixel pitch of the CCD camera was 5.2 µm after the crack travelled a certain distance. To analyze both the specimen unloading and loading, the crack-opening and crack-closure phases throughout the cycle were studied in this way to determine CTOD in every frame recorded. The CTOD load plot slopes were then used to distinguish and quantify the elastic and plastic contributions to the CTOD. 

**Figure 5:**
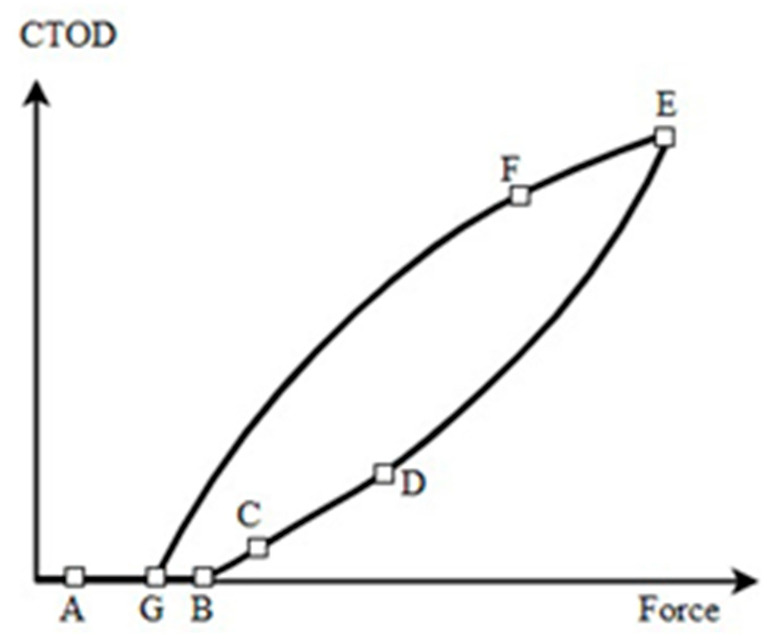
Crack-tip opening displacement versus load curve represented as a schematic, with the identification of characteristic points [83].

In addition to studying crack shutting, large- and small-scale yielding at the crack tips and fatigue propagation can also analyzed when tested with biaxial loading conditions by examining the relationship between the conventional plot curve of CTOD and the load. Moreover, this correlation can also be used to rule out elastic deformation, which is only weakly linked to the FCGR [81]. The methodology set out in this work is critical to efficiently investigate the CTOD–load relationship when using DIC-derived full-field displacement data [82]. Original displacement data can be used to create the CTOD versus load curves depicted in Figure 5 but only for CTOD_p_ because it produces permanent damage at the crack tip. 

Figure 5 depicts the data points retrieved from 2D-DIC as a standard CTOD versus load curve. Given the sensitivity of the technique, DIC identified very minor displacements between points A and B, even though the crack should be totally closed for loads. The increasing load caused opening of the crack at point B, which continued linearly up to point D, the elastic regime limit of the system. Plastic deformation gradually increased between points D and E, peaking at the maximum load. Load reduction causes reverse elastic deformation in line with the loading rate between points E and F. As a result, the maximal permissible deviation between the elastic deformation loading and unloading slopes was 1%. Reverse plastic deformation began after point F, with the crack closing again at point G. Of note, the loads for opening and closing also slightly differed. This property may be associated with events occurring at the closed crack tip and so it would be worth investigating further. 

**Figure 6:**
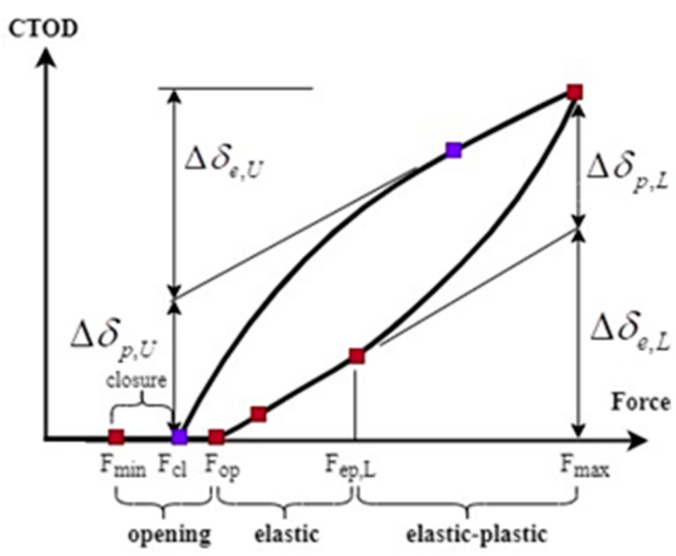
Parameters for fatigue-crack-growth analysis [83].

As set out in Figure 6, the minimum and maximum loads employed during the cycle were *F_min_* and *F_max_*. In turn, the loads present when the crack opened or closed during loading and unloading, respectively, were *F_B_* and *F_G_*, with these parameters corresponding to points B and G (Figure 5). 

These characteristics yielded two interesting crack attributes, *U_op_* and *U_cl_*, as set out in Equations (4) and (5) below.

Crack-opening level:

(4) $$U_{op}=\frac{F_{B}-F_{min}}{F_{max}-F_{min}}$$

Crack-closure level:

(5) $$U_{cl}=\frac{F_{G}-F_{min}}{F_{max}-F_{min}}$$

Finally, ${U_{op}\geq U}_{cl}$ was always valid for the CTOD versus load cycle curve.

### 3.2. Algorithm to Obtain Measurement Parameters

To ensure that the parameters were extracted correctly, a clearly outlined sequence of steps, based on a set of positions on the CTOD versus load cycle plot (Figure 6), must be adhered to. Point *F*_min_, representing the minimum load, gradually increases to a peak value, *F*_max_, and then returns to the minimum value. However, depending on the DIC methodology employed, the exact number of points on the CTOD versus load cycle plot may vary.

#### 3.2.1. Determination of Crack Opening and Crack Closure

**Figure 7:**
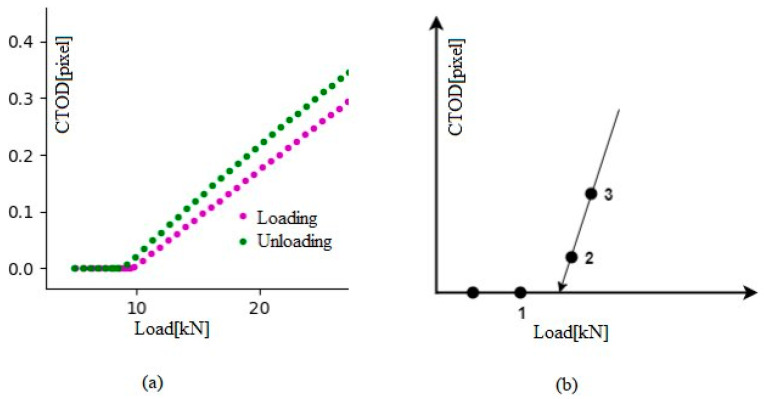
(**a**) Changes in the data near the crack-opening and crack-closing positions shown through selected plots of loading and unloading; (**b**) a graphic representation showing the procedure used to extrapolate the loads at which the crack opened and closed [83].

The levels of crack opening and closing are determined using Equations (4) and (5). Thus, in Figure 7a, the purple and green data points represent CTOD versus load cycle curve loading and unloading, respectively. The extrapolation procedure is represented as a schematic in Figure 7b, which shows the application of loading in a finite number of steps, with crack closing at step 1 and crack opening at step 2. Consequently, the crack opens somewhere between points 1 and 2. To determine the actual opening load, two post-opening positions (points 2 and 3) were used to establish linear extrapolation. However, it is worth noting that this kind of extrapolation is not needed under plain strain conditions where the crack is already open.

The load at which the crack opened was sometimes unclear because of inherent data variability. This may have resulted from experimental noise derived from the CCD camera signal, lighting settings, the speckled surface of the specimen, or surface warping when under load. This could perhaps be addressed by taking the first of the four successive CTOD values as point 2 when these four values increase after zero (Figure 7b). 

#### 3.2.2. The Elastic Regime System Slope (S_eU_) While Unloading

**Figure 8:**
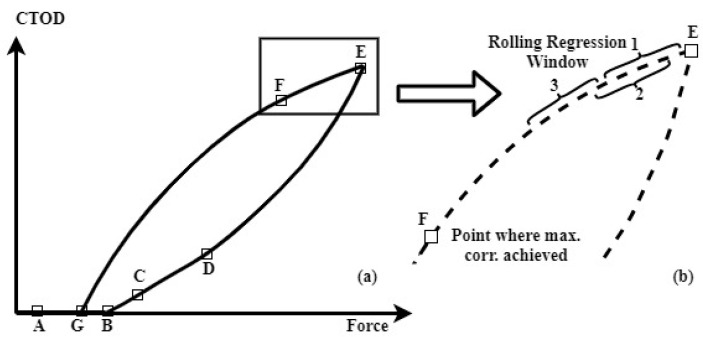
(**a**) Graphic representation demonstrating the application of *rolling regression* to maximize the point of correlation on the unloading curve. (**b**) Magnification of the curve in the maximum load. The numbers 1, 2, 3 are showing progressive steps (windows) taken by the rolling regression.

Next, the slope of the elastic regime system could be identified during the unloading phase (region EF in Figure 8a), where there was no interference from other events, such as crack closure, starting from the maximum cycle load. ‘Correlation coefficient maximization’ was used to determine how many points to consider to characterize the elastic regime slope. In this technique, the least-squares correlation coefficient is calculated, starting with the origin (the point of maximum load) and the second most maximal load point. The correlation coefficient is then recalculated by incorporating the next data point and discarding the origin data point, repeating the process with *rolling regression* for all the unloading data (Figure 8b). The *rolling regression* method available from the Python libraries [84] is employed to maximize the correlation coefficient. The *rolling regression* is a least-squares fitting that is independently applied to a fixed number of data points (or window), and then *rolls* or moves across the rest of the data set. In this work, the window incorporated four data points. The data that produce the maximum correlation coefficient establish the very last point used to define the linear regime slope (point F, Figure 8).

#### 3.2.3. The Ranges of Plastic and Elastic Crack-Tip Opening Displacement during Unloading

Thus, based on the above, CTOD_e_ could be calculated by applying Equations (6) and (7), set out below.

(6) $$CTOD_{e}=S_{eU}\times F_{U}$$

In Equation (6), *S_eU_* represents the slope in the elastic part and *F_U_* signifies the unloading force. The difference between the maximum load (*F_max_* = *F_E_*) and the closing load (*F_G_*) was the maximum value of *F_U_*, while the plastic CTOD was the elastic values subtracted from the total value.

(7) $$CTOD_{p}=CTOD-CTOD_{e}$$

In FCG analysis, one of the most important factors, CTOD_p_ (δp), is zero in the elastic regime and gradually increases as unloading progresses. Thus, unloading provides a convenient way to quantify the elastic and plastic ranges throughout the course of a single cycle, with these being denoted as ∆*δe*_,*U*_ and ∆*δp*_,*U*_, respectively. Both ∆*δe*_,*U*_ and ∆*δp*_,*U*_ can be calculated as illustrated in Figure 6.

#### 3.2.4. Analysis of the Slope during Loading Portion of the Cycle (*S_e_*_,*L*_)

Subsequently, the slope of the elastic linear section was determined throughout the loading period (section CD, Figure 5), which only begins when the crack is completely open. After opening, given that the maximum permitted variation does not typically exceed 1%, if the slope of the two points significantly differed from *S_eU_*, point one was discarded and the next point in the analysis was examined. The operation continued until point *S_eU_* ± *tol* was reached on the slope, with *tol* representing the tolerance of the slope. Consequently, point C (Figure 5), is the first post-crack-opening data point that satisfies the elastic loading regime-deviation criterion.

#### 3.2.5. Elastic and Plastic Crack-Tip Opening Displacement throughout Loading

The CTOD_e_ and CTOD_p_ loading contributions were computed based on the loading-phase slope, *S_e_*_,*L*_, as described in Equation (8).

(8) $$CTOD_{e}=S_{e,L}\times \left(F-F_{C}\right)$$

The elastic and plastic CTOD ranges were as follows in Equations (9) and (10), respectively:

(9) $$\Delta \delta_{e,L}=S_{e,L}\times \left(F_{max}-F_{C}\right)+\delta_{C}$$

(10) $$\Delta \delta_{p,L}=CTOD_{\max\;}-\Delta \delta_{e,L}$$

where *∆δ_e_*_,*L*_ is the total CTOD_e_ during loading and *δ_C_* denotes a part of it that depends on where CTOD is measured. In addition, it is worth noting that variation between B and C may be non-linear.

## 4. Results and Discussion

**Figure 9:**
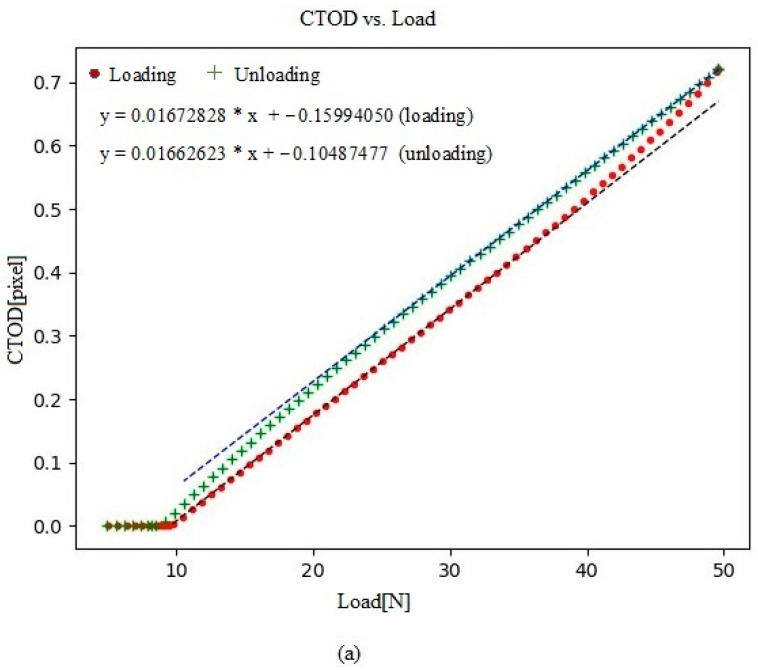

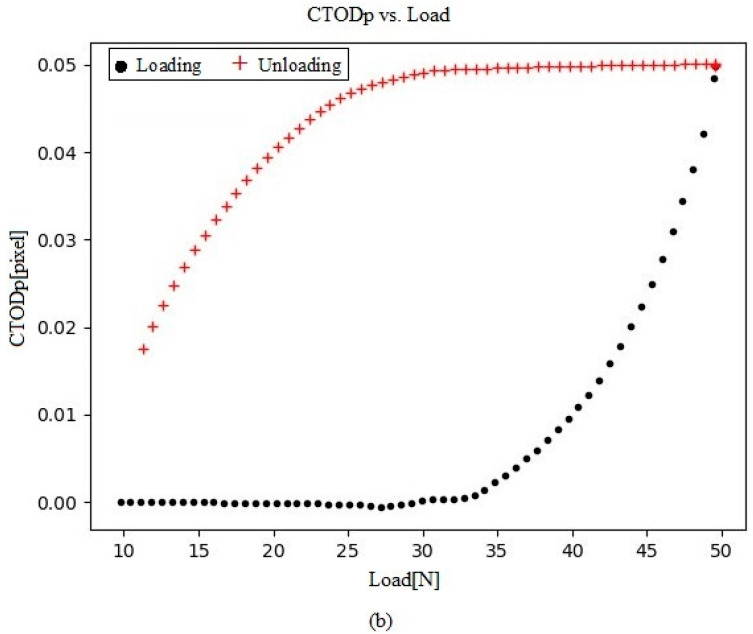
(**a**) The loading and unloading curves of crack-tip opening displacement (CTOD) plotted against load; (**b**) the plastic contribution of CTOD (CTOD_p_) plotted against load.

To facilitate comparisons, five sets of data depicting CTOD in relation to load were collected at five distinct locations behind the crack tip. After considering all of them, a satisfactory compromise 104 µm behind the crack tip was established. This optimized distance was (i) sufficiently close to the crack tip to be sensitive to its mechanics but (ii) not too far away to maintain an acceptable signal-to-noise ratio. For this purpose, several built-in libraries were used to create software in Python (v3.7), and matplotlib packages were used to graphically represent the data retrieved (Figure 9).

**Figure 10:**
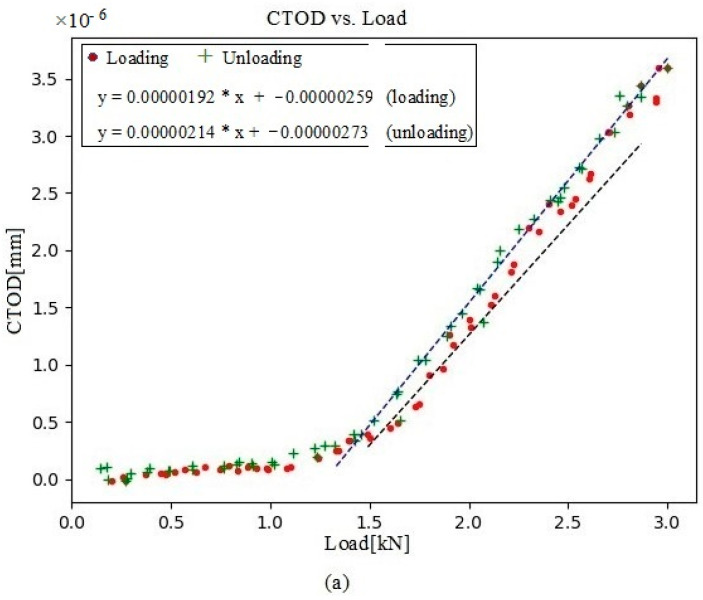

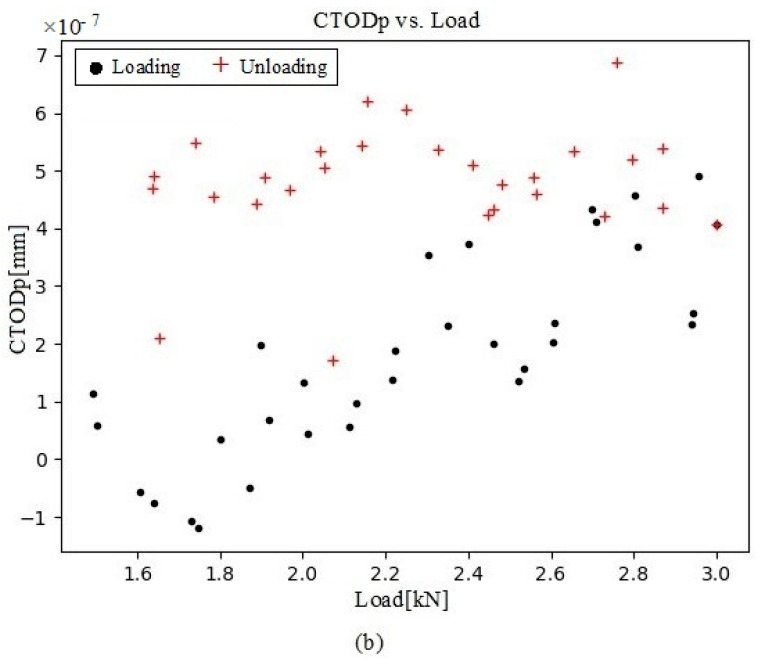
(**a**) Evolution of CTOD versus load at 230,792 cycles, together with the linear fitting for both portions of the cycle (loading and unloading); (**b**) the plastic contribution of the CTOD based on the information extracted from (**a**).

**Figure 11:**
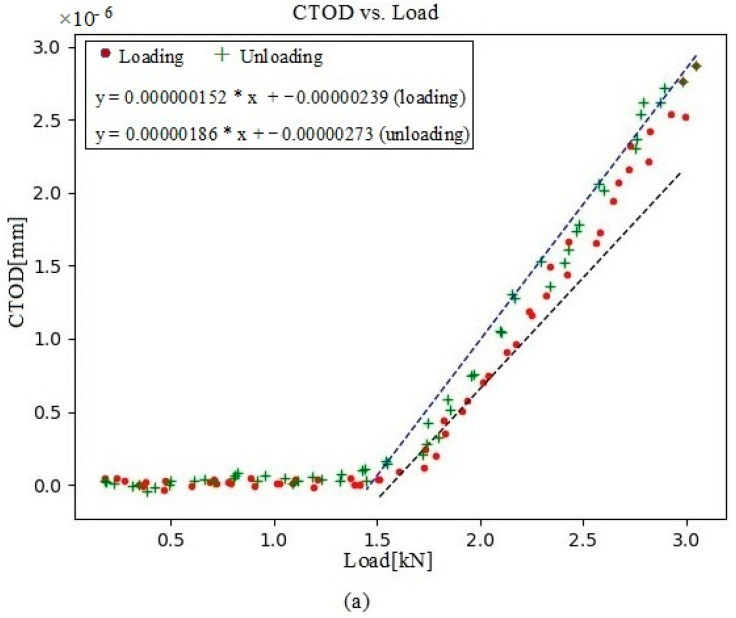

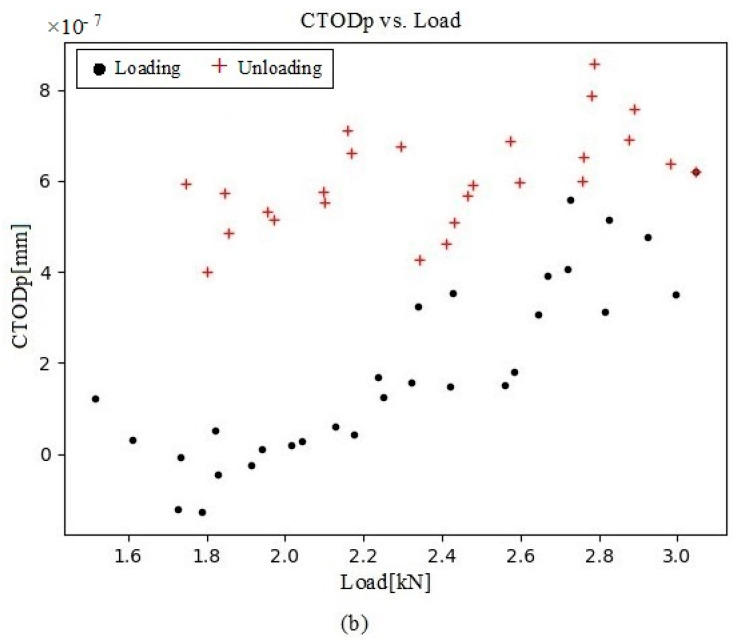
(**a**) Evolution of CTOD versus load at 250,786 cycles, together with the linear fitting for both portions of the cycle (loading and unloading); (**b**) the plastic contribution of the CTOD based on the information extracted from (**a**).

**Figure 12:**
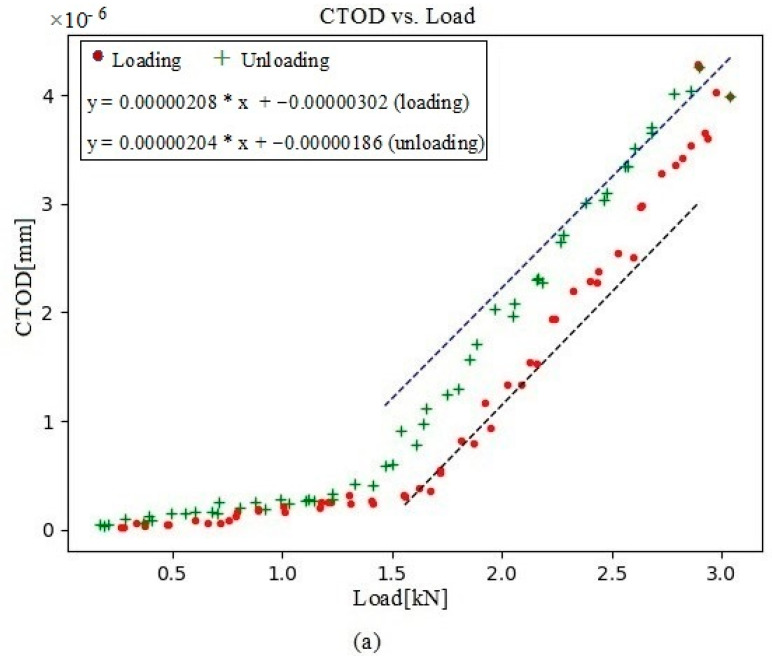

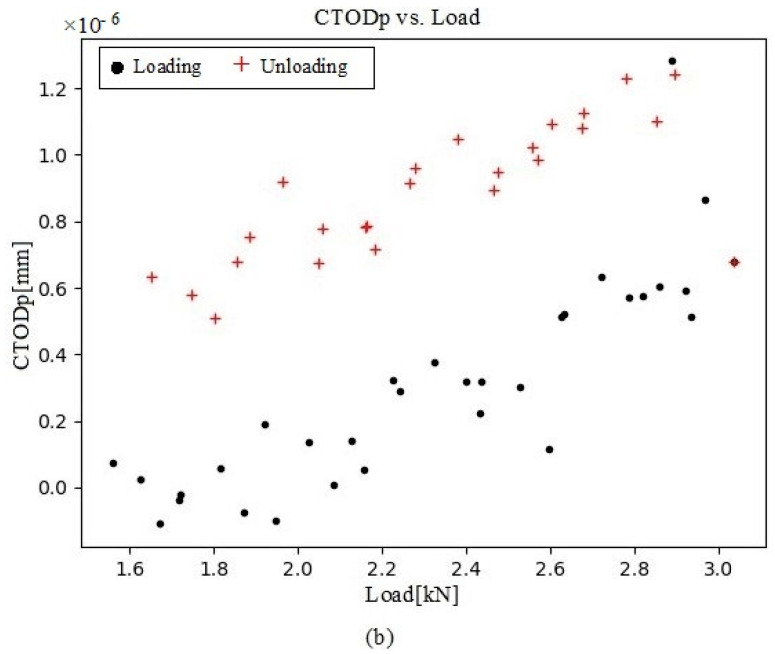
(**a**) Evolution of CTOD versus load at 290,838 cycles, together with the linear fitting for both portions of the cycle (loading and unloading); (**b**) the plastic contribution of the CTOD based on the information extracted from (**a**).

**Figure 13:**
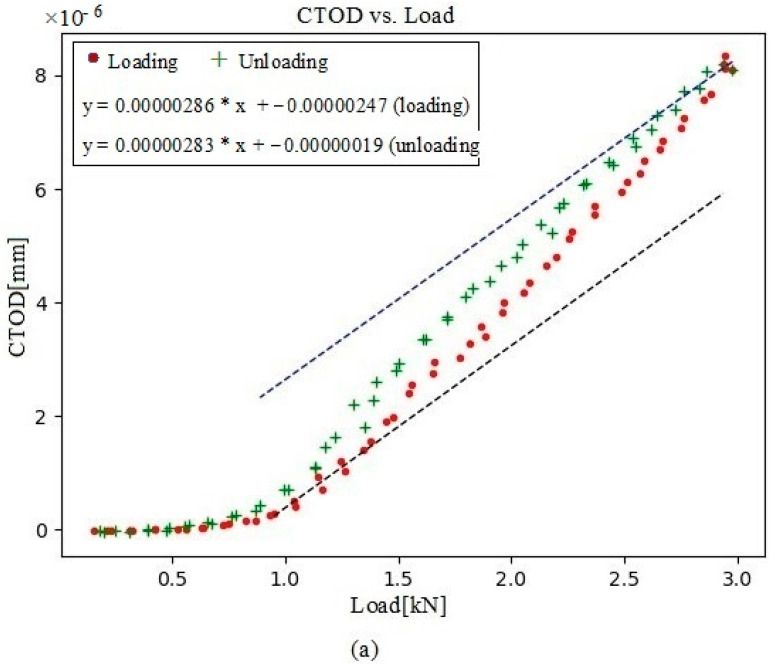

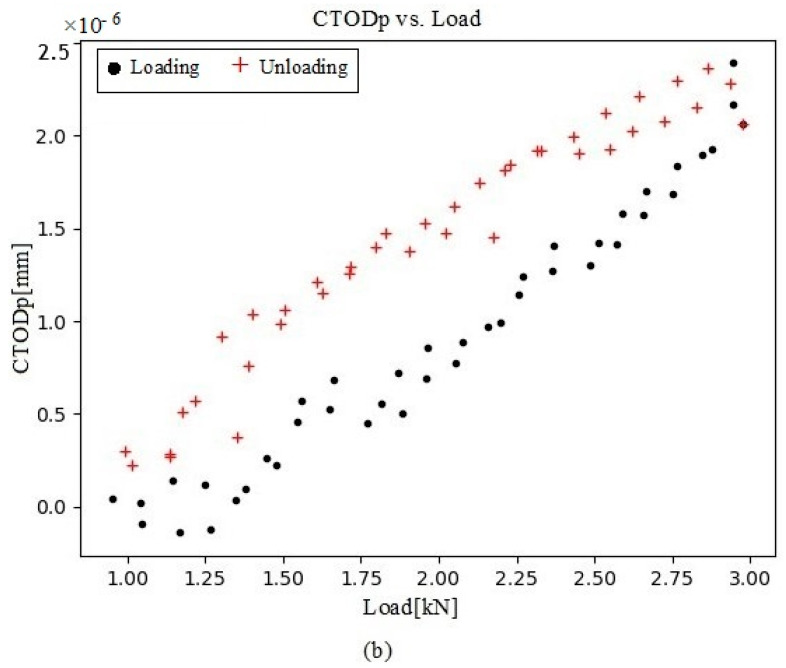
(**a**) Evolution of CTOD versus load at 331,273 cycles, together with the linear fitting for both portions of the cycle (loading and unloading); (**b**) the plastic contribution of the CTOD based on the information extracted from (**a**).

In this present analysis, the CTOD values versus load were acquired at four crack-propagation stages, 104 µm behind the crack tip. First, a data set was collected at 230,792 cycles, with a visible point of inflection (Figure 10a), indicating that contact with the crack was maintained for the first few loading values. CTOD_p_ was depicted for both the loading and unloading plots (Figure 10b). At 250,786 cycles, the crack contact data and the cyclic CTOD_p_ phase (Figure 11) were nearly identical to those for 230,792 cycles (Figure 10). In turn, the data collected at 290,838 cycles showed a shallower point of inflection (Figure 12), with this being indicative of less crack closure, resulting in larger CTOD_p_ values. With more crack progression, the last data set was collected at 331,273 cycles and showed much less crack closure, leading to higher CTOD_p_ values (Figure 13).

**Figure 14:**
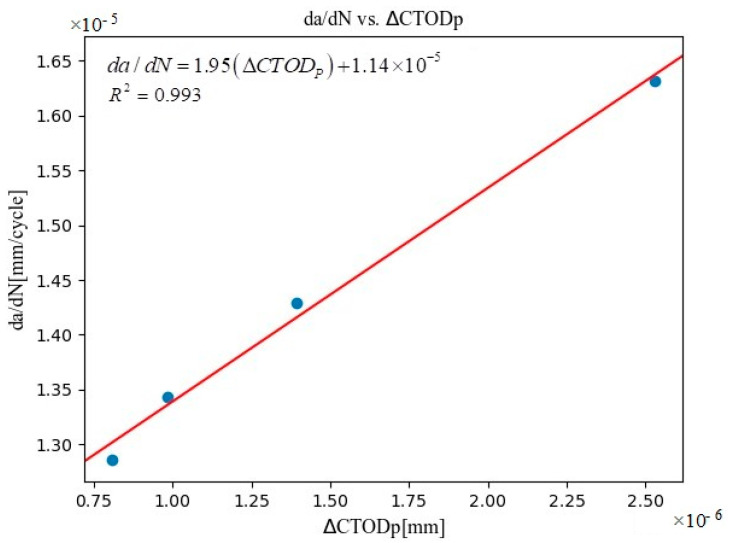
A graphic plot of da/dN versus change in plastic contribution of crack-tip opening displacement (∆CTOD_p_) for the data shown in Table 1. The blue dots represent the experimental points obtained with the new methodology.

In general, changes in the crack-opening load can be attributed to (i) the roughness of the fracture surface, with the effect being greater around the threshold values and tending to diminish with increasing crack length [85]; and (ii) the amount of material remaining at the front of the crack (the less the volume of material present, the lower its ability to compress the crack and keep it closed, meaning that the crack can open with a lower applied force) [86]. In other words, CTOD_p_ was significantly affected by the range of the load, especially by crack closure. In a similar manner to the SIF, the use of CTOD_p_ is based on its evolution per cycle. Accordingly, the range of CTOD_p_ (that is, ΔCTOD_p_) is proposed as the driving force. For each cycle, ΔCTOD_p_ is computed as the maximum CTOD_p_ measured minus the minimum CTOD_p_ measured. The primary results of this study, namely crack propagation across multiple fatigue stages, were based on the analysis of da/dN versus ΔCTOD_p_, as summarized in Table 1 and represented in the scatter plot shown in Figure 14. 

It is reasonable to assume that when a fatigue crack grows, CTOD_p_ (plastic deformation of the crack tip) also increases. Thus, Python NumPy [84] linear regression was used to fit a model to the data plot, which gave rise to the expression in Equation (11), in which da/dN and ∆CTOD_p_ represent mm/cycle and mm, respectively.

(11) $$da/dN=1.95\left(\Delta CTOD_{P}\right)+1.14\times 10^{-5}$$

The goodness-of-fit of this expression to the experimental data was high, as shown by the linear-model-fitting residual value (R^2^: 0.993). Furthermore, the linear relationship between da/dN and ∆CTOD_p_ was extremely intriguing because it means that the dimensional concerns with standard da/dN–∆K curves can be avoided. The slope of this linear relationship can be viewed as a material attribute, while its variation may rely on the method employed (i.e., the distance behind the crack tip where the measurements are taken or the geometry of the specimen). In this experimental set-up, the specimen thickness used was 12 mm and dominance of the plain strain was assumed. In order to be able to predict the fatigue-crack-growth behavior of 316L stainless steel, more tests are required, and the analysis needs to be extended to more samples, including different thicknesses, different fatigue geometries, and additional crack length ranges. Of note, the aforementioned linear relationship implies that the stress state did not change during the experiment. This work also emphasized the power of applying the DIC approach at the submicron level while still managing to achieve good spatial resolution.

## 5. Conclusions

This research used a new tool to experimentally investigate crack growth in 316L austenitic stainless steel. This algorithm, written in Python, correlates full-field digital images to isolate the plastic contribution of CTOD to quickly extract characteristic points from CTOD_p_ versus load data correlations. Some key data output by this tool are the crack-opening and crack-closure levels, elastic and plastic contributions of CTOD, and loading and unloading curve ranges. Given that crack-tip plasticity was the most significant factor in fatigue crack growth, the relationship between da/dN and ∆CTOD_p_ could perhaps be employed to forecast crack growth resulting from fatigue. The new tool is expected to be valid on other metal alloys that are homogeneous and isotropic. Of note, in this work, the relationship between ∆CTOD_p_ and the propagation of fatigue cracks in 316L stainless steel was shown to be linear. CTOD_p_ was used to derive a crack-propagation law, which naturally incorporated phenomenon, including residual stresses and crack closing, that directly affect crack-tip plasticity. The advantages of using ∆CTOD_p_ rather than ∆K are that (i) both FCGRs and CTODs are lengths and so the slope is unitless and can be considered a material property; and (ii) the relationship between CTOD_p_ and da/dN is linear, rather than being logarithmic as in the case of da/dN and ∆K. 

## Figures and Tables

**Table 1:** The change in the displacement of the plastic component of crack-tip opening displacement (ΔCTOD_p_) and fatigue crack growth da/dN data with increasing fatigue cycles.

Measurement Stages	N (Accumulated Cycles)	∆CTOD_p_ (mm)	da/dN (mm/Cycle)
1	230,792	8.06564 × 10^−7^	1.28577 × 10^−5^
2	250,786	9.84161 × 10^−7^	1.34326 × 10^−5^
3	290,838	1.39211 × 10^−6^	1.42913 × 10^−5^
4	331,273	2.53057 × 10^−6^	1.63225 × 10^−5^

## Data Availability

Raw data of this article are available upon request from the authors.

## Nomenclature

*F_cl_*	closure load
*F_ep,L_*	ela stic–plastic transition load
*F_max_*	maximum load
*F_min_*	minimum load
*F_op_*	opening load
*F_U_*	force applied during unloading
*S_e,L_*	loading slope (elastic contribution)
*S_e,U_*	unloading slope (elastic contribution)
∆δ_e,L,_ ∆δ_p,L_	range of CTOD elastic and plastic contributions during loading
∆δ_e,U,_ ∆δ_p,U_	range of CTOD elastic and plastic contributions during unloading
*U_op_*	crack-opening level
*U_cl_*	crack-closure level
